# Cholesterol Prevents Hypoxia-Induced Hypoglycemia by Regulation of a Metabolic Ketogenic Shift

**DOI:** 10.1155/2019/5829357

**Published:** 2019-09-11

**Authors:** Naama Miron, Oren Tirosh

**Affiliations:** Institute of Biochemistry, Food Science and Nutrition, The RH Smith Faculty of Agriculture, Food and Environment, The Hebrew University of Jerusalem, POB 12, 7610001 Rehovot, Israel

## Abstract

Blood cholesterol levels have been connected to high-altitude adaptation. In the present study, we treated mice with high-cholesterol diets following exposure to acute hypoxic stress and evaluated the effects of the diets on whole-body, liver glucose, and liver fat metabolism. For rapid cholesterol liver uptake, 6-week-old male C57BL/J6 mice were fed with high-cholesterol/cholic acid (CH) diet for 6 weeks and then were exposed to gradual oxygen level reduction for 1 h and hypoxia at 7% oxygen for additional 1 hour using a hypoxic chamber. Animals were than sacrificed, and metabolic markers were evaluated. Hypoxic treatment had a strong hypoglycemic effect that was completely blunted by CH treatment. Decreases in gluconeogenesis and glycogenolysis as well as an increase in ketone body formation were observed. Such changes indicate a metabolic shift from glucose to fat utilization due to activation of the inducible nitric oxide synthase/AMPK axis in the CH-treated animals. Increased ketogenesis was also observed in vitro in hepatocytes after cholesterol treatment. In conclusion, our results show for the first time that cholesterol contributes to metabolic shift and adaptation to hypoxia in vivo and in vitro through induction of HIF-1*α* and iNOS expression.

## 1. Introduction

Molecular oxygen, as the final electron accepter in aerobic respiration, is essential for the survival of most metazoans. To adapt to oxygen deprivation (hypoxia), multicellular organisms have evolved complex networks that regulate metabolic changes at both systemic and cellular levels to mediate changes in angiogenesis/vascular remodeling, glycolytic metabolism, and cell proliferation. Diminished oxygen supply requires metabolic adaptation to cope with the resulting energetic stress and to maintain the supply of ATP [1]. Diminished oxygen levels decrease utilization of the electron transport chain in the mitochondria and will shift mitochondrial oxygen reduction to water in the direction of nitrite reduction to nitric oxide to induce vasodilatation and increase blood supply [2]. Additional adaptations to hypoxic stress could be due to changes in gene expression under the control of hypoxia-inducible factor 1-*α* (HIF-1*α*) [3], a master regulator that mediates most transcriptional changes during hypoxia. Adaptations could also be due to more efficient use of substrates during acute hypoxia [4]. The common denominator in all such mechanisms is facilitation of glucose utilization via anaerobic and aerobic metabolism.

HIF-1*α* activity has also been linked to lipid metabolism via hypoxia-inducible protein 2 (HIG2). HIG2 is located at the hemimembrane of lipid droplets (LDs) and colocalizes with the LD proteins adipophilin and TIP47 [5]. HIG2 overexpression under normoxic conditions increases neutral lipid deposition in macrophages and stimulates cytokine expression [6]. HIG2 is detected in atherosclerotic arteries and in patients with fatty liver disease, suggesting that this product of the ubiquitously inducible HIF-1*α* gene target may play an important functional role in disease progression and etiology associated with ectopic lipid accumulation [6–8]. Exposure of human cells to hypoxia reportedly causes triglyceride accumulation and LD formation [9].

The effect of human diets on metabolic adaptation to hypoxia is unknown. Interestingly, acclimatization to high altitudes and induction of erythropoiesis increase serum cholesterol levels. Mean hematocrit was reported to be significantly higher at high altitudes, as was mean serum cholesterol [10]. High-density lipoprotein cholesterol levels were reported to increase linearly and significantly at high altitudes [11]. Oxygen therapy was demonstrated to decrease both total cholesterol and low-density lipoprotein cholesterol. Indeed, serum cholesterol has been shown to decrease after initiation of home oxygen therapy [12]. The effects of low-carbohydrate and high-fat diets, especially those with high cholesterol, on metabolic adaptation and response to hypoxia should be evaluated due to the growing trend of low-carb diets, such as ketogenic and paleo diets, among others. It is important to understand the connections between nutritional status and metabolic adaptations to environmental conditions such as hypoxia.

We previously reported a connection between HIF-1*α* activation and dietary cholesterol [13]. Additionally, we demonstrated that cholesterol facilitates HIF-1*α* stabilization under normoxic conditions both in vivo and in vitro [13, 14]. The atherogenic diet is an accelerated model for cholesterol loading into animal liver tissue [15, 16] and is an excellent model for evaluating the metabolic and pathological effects of dietary cholesterol. The aim of the current study was to treat mice with high-cholesterol diets following exposure to acute hypoxic stress and to evaluate the effects of such diets on whole-body, liver glucose, and liver fat metabolism. The suggested mechanism by which cholesterol contributed to hypoxic adaptation and survival may possibly allow future development of therapeutic strategies for prevention of hypoxic damage prior to liver transplantation and other hypoxic conditions.

## 2. Materials and Methods

### 2.1. Animal Experiments

Forty C57BL/6J mice aged 4 weeks were purchased from Harlan Laboratories (Jerusalem, Israel) and kept in a controlled environment (22–24°C) with a 12 h light/dark cycle and free access to food and water. All procedures were performed in accordance with the Institutional Animal Care and Use Committee (IACUC) of the Hebrew University of Jerusalem (approval no. AG-16-14907-4; Jerusalem, Israel).

**Figure 1:**
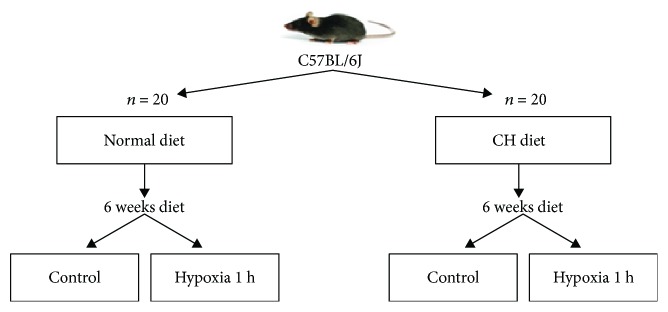
Design of in vivo experiment.

Mice were divided into two equal experimental groups (5 mice per cage) such that the average weights of cages and groups would not be significantly different. Mice were fed with one of the following diets: normal diet or high-cholesterol diet (CH diet, normal diet +1% cholesterol +0.5% cholate) (Figure 1). The composition of the diets is shown in [Supplementary-material supplementary-material-1].

Body weights were recorded weekly. At the end of the feeding period (6 weeks), following an overnight fasting, half of the mice in each group were placed in a hypoxic chamber (Coy 8430215; Coy Laboratory Products Inc., Grass Lake, MI, USA) with 7% oxygen for 1 h (following a gradual decrease in oxygen level over 1 h).

At the end of the experiment, the mice were sacrificed, and blood as well as liver and testicular adipose tissue were collected for analyses. Additional liver samples were fixed in 4% paraformaldehyde and embedded in paraffin for histological examination.

### 2.2. Cell Culture and Treatments

Mouse transformed primary hepatocytes were maintained at 37°C under 5% CO_2_ in Dulbecco's modified Eagle's medium (DMEM) supplemented with 10% FBS, penicillin (100 U/ml), and streptomycin (100 mg/ml).

Cells were incubated with or without water-soluble cholesterol (WSC) (0.2 mg/ml; Sigma-Aldrich, St. Louis, MO, USA) for 6 h. Half of the cells in each group were subjected to hypoxia for either 1 or 6 h. For hypoxic exposure, culture dishes were placed in a modular incubator chamber (Billups-Rothenberg, Inc., Del Mar, CA, USA) that was flushed with <1% O_2_/5% CO_2_/balanced N_2_ and incubated at 37°C [17].

### 2.3. Blood Parameters and Biochemical Analyses

Levels of the liver blood enzymes serum alanine aminotransferase (ALT/SGPT) and serum aspartate aminotransferase (AST/SGOT) were measured using an automated clinical chemistry analyzer along with cholesterol and TGs analyses (American Laboratories Ltd., Omaha, NE, USA). Fasting glucose levels were measured in tail-tip blood using an Optium Xceed glucometer (Abbott Laboratories Inc., Abbott Park, IL, USA).

### 2.4. Liver Histology

Paraffin molds were cut into 5 *μ*m sections and stained with hematoxylin-eosin (H&E) (performed by Patho-Lab Diagnostics Ltd., Ness Ziona, Israel).

### 2.5. Glycogen Analysis

Total glycogen in mouse livers was determined by enzymatic hydrolysis using *Aspergillus niger* amyloglucosidase (E-AMGDF; Megazyme, Bray, Ireland). Liver tissue (100 mg) was homogenized in 1 ml double-distilled water and boiled for 10 min to inactivate enzymes in the sample. The samples were centrifuged for 10 min at 18,000 x g (4°C), and the supernatants were collected. As a standard for the assay, 0.05–0.8 *μ*g/ml glycogen was used. Next, 3.5 units of amyloglucosidase were added to 10 *μ*l of each sample and standard, and their volumes were brought to 500 *μ*l with double-distilled water. The samples were incubated for 10 min at 55°C, and D-glucose was analyzed using a D-glucose assay kit (K-GLUC 10/15; Megazyme) according to manufacturer's instructions.

### 2.6. *β*-Hydroxybutyrate (*β*-HB) Levels

Generation of *β*-HB (a ketone body) was measured using a *β*-HB Colorimetric Assay Kit (700190; Cayman Chemical Company, Ann Arbor, MI, USA). *β*-HB levels were evaluated in liver tissues and serum samples of mice and in cell culture according to the manufacturer's instructions [18].

### 2.7. Protein Extraction and Western Blot Analysis

Whole-cell lysates were prepared in RIPA buffer. One hundred micrograms of the protein samples was separated by SDS-PAGE (7.5%), transferred to nitrocellulose membranes, and subjected to western blotting as previously described. Blots were probed with a primary antibodies specific to iNOS (Abcam, Cambridge, UK), ACC, p-ACC, AMPK, and p-AMPK (Cell Signaling Technologies, Danvers, MA, USA). Secondary antibodies were obtained from Jackson ImmunoResearch (West Grove, PA, USA). Results were normalized to the intensity of the *β*-actin gene (Abcam).

### 2.8. Quantitative Real-Time Polymerase Chain Reaction

Total RNA was isolated using the TRIzol reagent (Sigma-Aldrich) according to the manufacturer's protocol. Complementary DNA was prepared using the High-Capacity cDNA reverse transcription kit (Applied Biosystems, Foster City, CA, USA). Real-time PCR was performed with SYBR Green fluorescence using a 7300 Real-Time PCR System (Applied Biosystems) with specific primers. Primer sequences are presented in [Supplementary-material supplementary-material-1]. Fold changes in gene expression were determined relative to 18S mRNA expression.

### 2.9. Cellular Glucose Production Assay

Following overnight serum starvation, culture medium was replaced with glucose production medium, and cells were subjected to different treatments. Glucose secreted by cells was measured using a D-glucose assay kit (K-GLUC 10/15, Megazyme) [14].

### 2.10. Cell Viability

Loss of cell membrane integrity was detected by propidium iodide (PI) staining. After treatment, the cells were trypsinized, filtered through a 90 *μ*m mesh grid, stained with PI (2 *μ*g/ml), and measured by flow cytometry (FACSCalibur; BD Biosciences, Franklin Lakes, NJ, USA) with the fluorescence settings of excitation at 488 nm and emission at 575 nm. Data were collected from 10,000 cells.

### 2.11. Statistical Analysis

Significance was determined using an independent one-way ANOVA test with Tukey-Kramer post hoc analysis. Descriptive statistics are expressed as means ± SE. Means with different letters are statistically different, *P* < 0.05. a was indicated to the highest means and b, c, d, etc. to lower values of means, respectively.

The significance of the differences between means was determined by Student's *t*-test when a single comparison was performed. Differences were considered significant at *P* < 0.05.

JMP version 14.0.0 (SAS Institute, Chicago, IL USA) was used for all analyses.

## 3. Results

### 3.1. Cholesterol Promotes Adipose Tissue Lipolysis and Liver Enlargement

**Figure 2:**
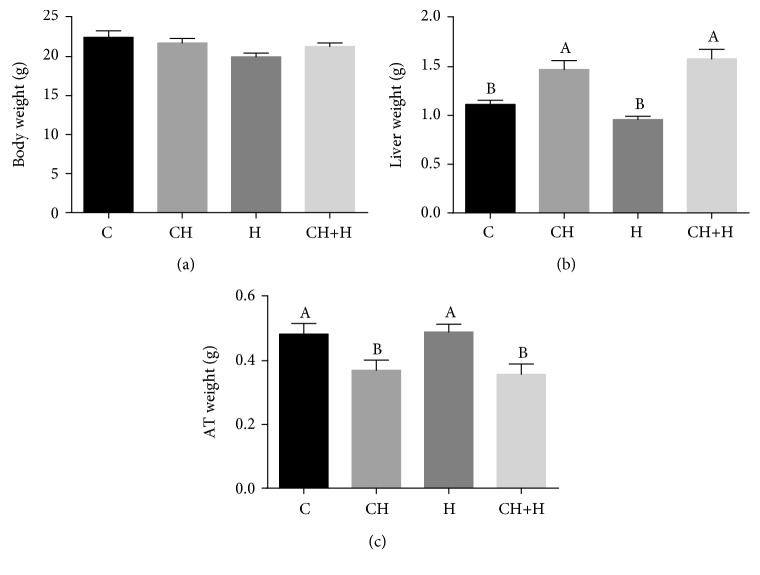
Effect of CH diet (CH), hypoxia (H), or their combination (CH+H) compared to the control (C) on (a) body weight, (b) liver weight, and (c) adipose tissue (AT) weight. Values are expressed as mean ± SE (*n* = 8–10 for each group). Means with different letters are statistically different, *P* < 0.05.

As reported previously, the CH diet promoted hepatic enlargement in mice [14] over control levels. Supplementation with CH diet resulted in a fatty liver phenotype ([Supplementary-material supplementary-material-1]). In groups fed the CH diet, total body weight at the end of the experiment was not significantly different than that in controls (Figure 2(a)). The CH diet caused a significant increase in liver weight compared to that in controls, while short-term hypoxia had no effect (Figure 2(b)). The CH diet with or without hypoxia treatment resulted in decreased testicular adipose tissue mass, an indicator of increased lipolysis [19] (Figure 2(c)). This indicates fatty acids flux from adipose tissue to the liver.

### 3.2. Glucose and Fat Metabolism Is Affected by Cholesterol under Hypoxic Conditions

**Figure 3:**
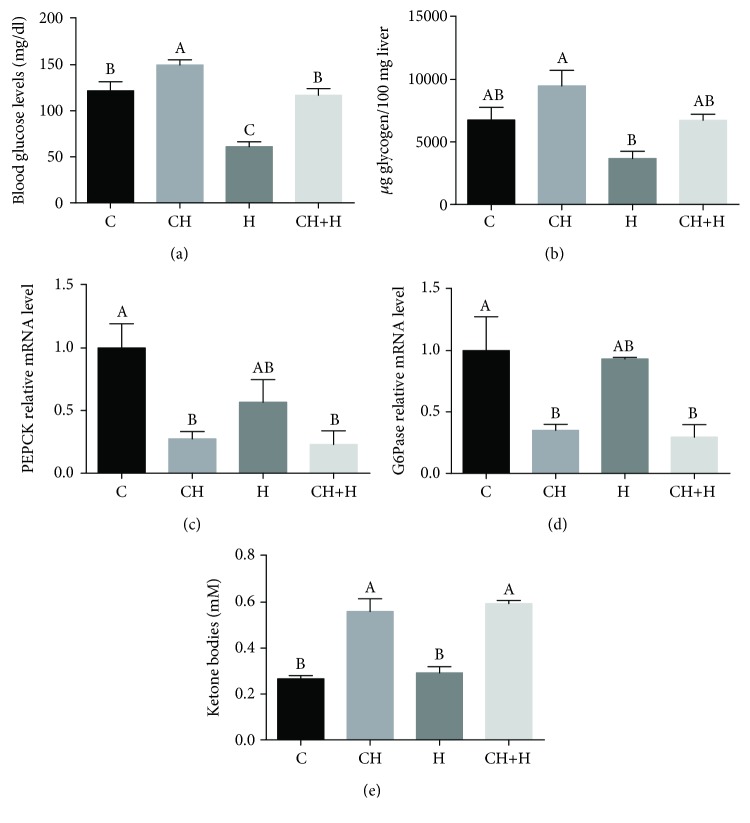
Effect of CH diet (CH), hypoxia (H), or their combination (CH+H) compared to the control (C) on (a) blood glucose levels, (b) liver glycogen content, (c) liver mRNA levels of *PEPCK*, (d) liver mRNA levels of *G6Pase*, and (e) liver ketone body levels. Values are expressed as means ± SE (*n* = 8–10 for each group). Means with different letters are statistically different, *P* < 0.05.

We exposed the mice to hypoxic conditions after supplementation with the CH diet. Following 6 weeks of supplementation with CH diet, an increase in serum cholesterol was observed with a concomitant decrease in serum triglyceride levels ([Supplementary-material supplementary-material-1]). Hypoxia caused hypoglycemia (normal blood levels in mice is around 124 mg/dl [20]) in mice fed with the normal AIN93-M diet, while CH diet consumption protected against hypoxia-induced hypoglycemia (Figure 3(a)). While fasting, liver-generated glucose and ketone bodies provide essential metabolic fuel to extrahepatic tissues. Glucose released into circulation is derived from gluconeogenesis and glycogenolysis, while nonesterified fatty acids are oxidized in hepatic mitochondria through fatty acid *β*-oxidation and generate ketone bodies (ketogenesis) [21–23]. Therefore, we studied whether increased glycogenolysis is a possible pathway for prevention of hypoxia-induced hypoglycemia. Surprisingly, glycogen reservoirs in CH diet-fed mice were not depleted, while a significant decrease in liver glycogen was observed in mice exposed to hypoxia without cholesterol (Figure 3(b)).

To test whether the prevention of hypoxia-induced hypoglycemia by the CH diet resulted from enhanced gluconeogenesis, mRNA levels of the gluconeogenic genes *PEPCK* (Figure 3(c)) and *G6Pase* (Figure 3(d)) were analyzed. CH diet consumption decreased liver mRNA levels of these genes as compared to levels in control and hypoxia-stressed mice alone. Therefore, a possible explanation for the inhibition of glycogenolysis and gluconeogenesis alongside with higher blood glucose levels is less demand for glucose for energy production, indicating a possible metabolic shift from glucose to fat utilization.

To confirm this metabolic shift to fatty acid oxidation and ketogenesis, *β*-hydroxybutyrate (*β*-HB) levels were analyzed in mouse livers (Figure 3) and also in another experiment in the serum ([Supplementary-material supplementary-material-1]). A significant increase in ketone body levels was observed in the livers of mice fed with the CH diet, while hypoxia had no effect (Figure 3(e)).

### 3.3. Effect of Cholesterol on Fatty Acid Oxidation Pathways

**Figure 4:**
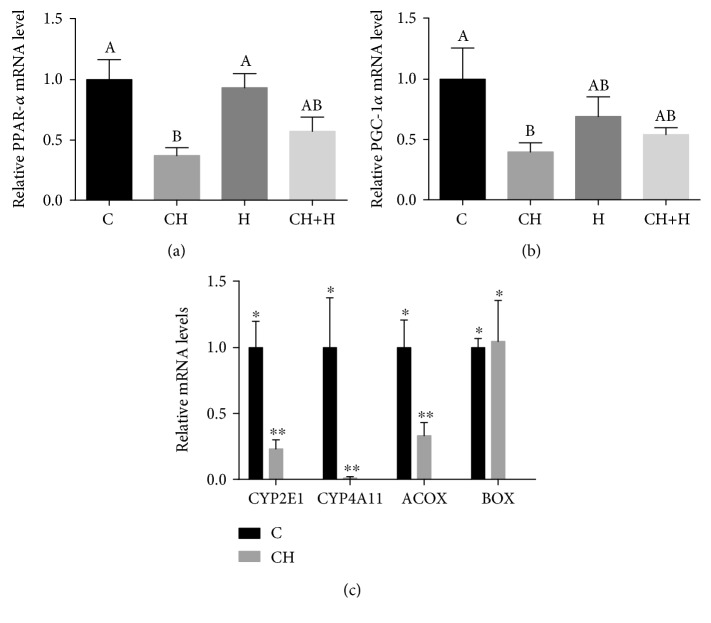
Effect of CH diet (CH), hypoxia (H), or their combination (CH+H) compared to the control (C) on liver mRNA levels of (a) PPAR-*α* and (b) PGC-1*α*. Values are expressed as means ± SE (*n* = 6 for each group). Means with different letters are statistically different, *P* < 0.05 (a, b). (c) Effect of CH diet (CH) compared to the control (C) on liver mRNA levels of CYP2E1, CYP4A11, ACOX, and BOX. The significance of the differences between means was determined by Student's *t*-test. Differences were considered significant at *P* < 0.05.

Ketone bodies are derived primarily from fatty acid oxidation [24]. The CH diet decreased liver mRNA levels of the key mitochondrial *β*-oxidation enzymes PPAR*α* (Figure 4(a)) and PGC-1*α* (Figure 4(b)).

When cytosolic fatty acids accumulate due to impairment of oxidative capacity in mitochondria, alternative pathways in the peroxisomes (*β*-oxidation) and in microsomes (*ω*-oxidation) are activated. In peroxisomal *β*-oxidation, straight-chain acyl-CoA oxidase (ACOX) and branched-chain acyl-CoA oxidase (BOX) are responsible for the initial oxidation of very long-chain fatty acyl-CoAs [25]. CH diet consumption markedly decreased liver mRNA levels of ACOX as compared to those in the control, while BOX mRNA levels were unaltered (Figure 4(c)).

In microsomal *ω*-oxidation, CYP2E1 and CYP4A11, which are inducible hepatic microsomal cytochrome P-450s, initiate the autopropagative process of lipid oxidation [25]. However, unexpectedly marked decrease in liver mRNA levels of CYP2E1 and CYP4A11 was detected in CH diet-fed mice compared to control levels (Figure 4(c)).

### 3.4. Activation of the AMPK Pathway by Cholesterol

**Figure 5:**
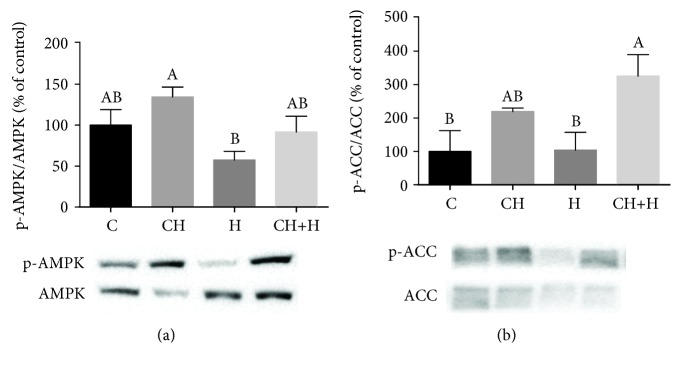
Effect of CH diet (CH), hypoxia (H), or their combination (CH+H) compared to the control (C) on (a) AMPK phosphorylation and (b) ACC phosphorylation. Values are expressed as means ± SE (*n* = 6–8 for each group). Means with different letters are statistically different, *P* < 0.05.

Since the CH diet did not appear to increase fatty acid oxidation by modulating gene expression, we reasoned that the mechanism for increased *β*-oxidation rate could be related to AMP-activated protein kinase (AMPK) activation [26]. Indeed, CH diet consumption increased AMPK activation in mouse livers. In addition, the decrease in AMPK activation caused by hypoxia was prevented by the CH diet (Figure 5(a)).

Activation of AMPK stimulates fatty acid oxidation and ketogenesis, among other pathways, through phosphorylation and inactivation of ACC [25]. Increased ACC phosphorylation was observed in the livers of CH diet-fed mice with and without hypoxia treatment (Figure 5(b)).

### 3.5. Connection between Cholesterol and Nitric Oxide/Hypoxia Signaling Pathway in Metabolic Regulation

**Figure 6:**
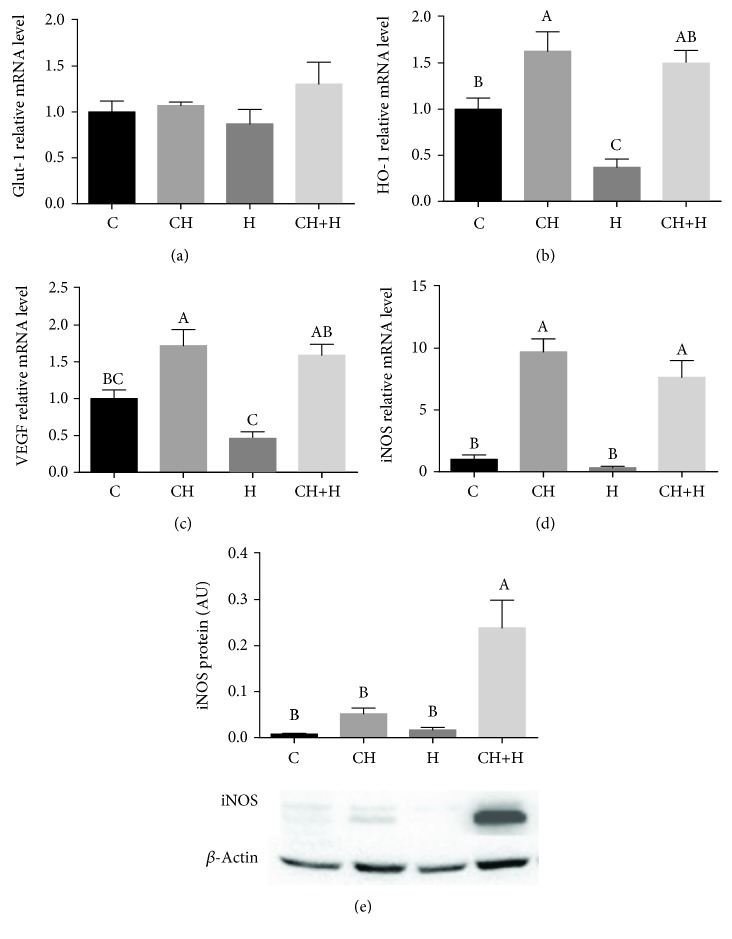
Effect of CH diet (CH), hypoxia (H), or their combination (CH+H) compared to the control (C) on liver mRNA levels of (a) *Glut-1*, (b) *HO-1*, (c) *VEGF*, and (d) iNOS and on (e) iNOS protein expression levels. Values are expressed as means ± SE (*n* = 8–10 for each group). Means with different letters are statistically different, *P* < 0.05.

HIF-1*α* transcriptional activity was estimated by induction of its target genes *HO-1*, *VEGF*, and *Glut-1*. A significant increase in liver mRNA levels of *HO-1* (Figure 6(b)) and *VEGF* (Figure 6(c)) was observed in CH diet-fed mice, while *Glut-1* mRNA levels were statistically similar among the different groups but with higher mean value compared to other treatments (Figure 6(a)). Previously, it was demonstrated that iNOS can increase GLUT1 expression in muscles by an NO-dependent mechanism [27] which can indicate better liver glucose export to prevent hypoglycemia. In relation to lipid metabolism, we previously demonstrated that iNOS is important to AMPK activation due to low activation in iNOS-deficient mice [14]. The association between induction of HIF-1*α* and iNOS and the protective effect of the CH diet against hypoxia-induced hypoglycemia was demonstrated.

The combined treatment of the CH diet and hypoxia exhibited a synergistic effect and caused a significant increase in iNOS protein expression, while the CH diet alone caused only a moderate increase, and hypoxia treatment alone had no effect (Figure 6(e)). Liver mRNA levels of iNOS were significantly increased in mice fed with the CH diet over levels in mice fed with a normal diet with or without hypoxia treatment (Figure 6(d)).

**Figure 7:**
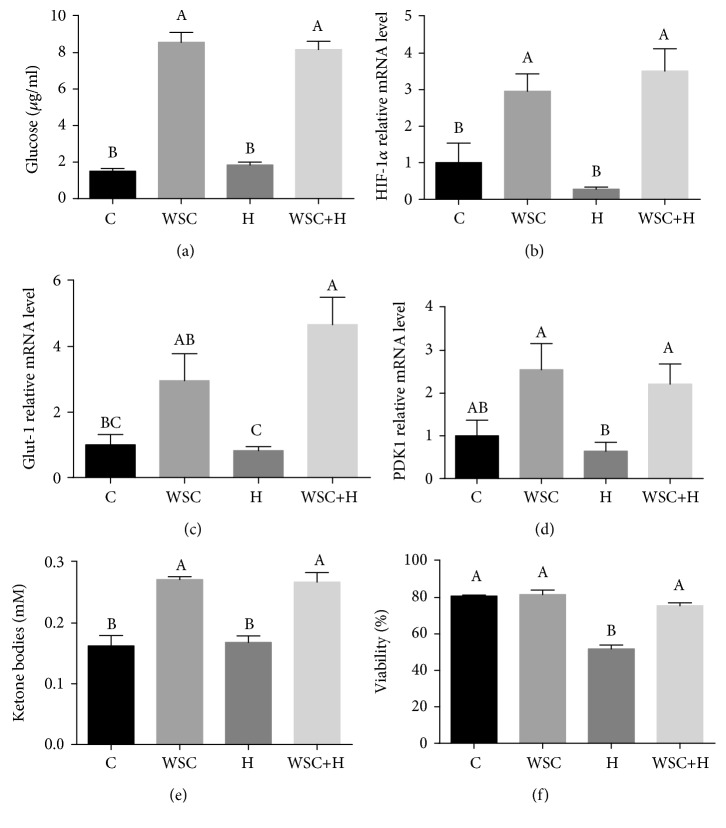
Effect of water-soluble cholesterol (WSC) treatment of hepatocytes, hypoxia treatment (H), or their combination (WSC+H) on (a) hepatocellular glucose release, (b) mRNA levels of HIF-1*α*, (c) mRNA levels of *Glut-1*, (d) mRNA levels of *PDK1*, (e) ketone body levels, and (f) cell viability. Values are expressed as means ± SE (*n* = 4–8 for each group). Means with different letters are statistically different, *P* < 0.05.

The effect of cholesterol on the liver metabolic response to hypoxia was further evaluated in vitro. Incubation of hepatocytes with water-soluble cholesterol (WSC) significantly increased glucose output, while hypoxia treatment had no effect (Figure 7(a)). Consistently with this increased output, mRNA levels of HIF-1*α* (Figure 7(b)) and its target genes *Glut-1* (Figure 7(c)) and *PDK1* (Figure 7(d)) were enhanced following cholesterol treatment of hepatocytes. Consistently with the in vivo results, a significant increase in ketone body levels was observed in hepatocytes treated with cholesterol with and without hypoxia (Figure 7(e)).

HIF-1*α* plays pivotal roles in cell survival during hypoxia. Moreover, the enhanced expression of *Glut-1* and *PDK1* mediated by HIF-1*α* in response to prolonged hypoxia represents a fundamental adaptation critical to the maintenance of hepatocellular homeostasis [28]. Cell viability following the different treatments was evaluated and indicated the protective effect of cholesterol against hypoxia-induced cell death (Figure 7(f)); hypoxia caused significant cellular death, while cholesterol provided a cytoprotective effect.

## 4. Discussion

Altogether, the results demonstrate protective effect of cholesterol against hypoxia-induced hypoglycemia. Cholesterol increased fat utilization and protected against cell death in correlation with activation of the AMPK/iNOS axis. Whenever hypoxia is sustained, there is a switch from aerobic metabolism to glycolysis, which is a poor metabolic alternative [26]. Thus, hypoxia rapidly depletes glucose from blood and tissues. This is the physiological reason that intense and inefficient exercise [29], ascent to high altitudes [30], and certain ischemic conditions such as hypoxic hepatitis [31] lead to rapid fatigue. The lipid energy reservoir is much larger than that of carbohydrates. Therefore, utilization of energy from lipids under hypoxic conditions (not only under aerobic conditions) confers a great physiological advantage. In the current study by using a model of atherogenic diet without excess dietary triglycerides (fat), we demonstrated that high-cholesterol diets can induce a metabolic shift from carbohydrate to lipid utilization.

Acute hypoxia treatment caused hypoglycemia in mice. The current investigation demonstrated that consumption of cholesterol-rich diets protected against hypoxia-induced hypoglycemia. Hypoglycemia is a risk factor for increased hospital mortality in critically ill patients [32–34] and is associated with deleterious biological effects, such as increasing the systemic inflammatory response, inducing neuroglycopenia, inhibiting corticosteroid responses to stress, impairing sympathetic system responsiveness, and promoting cerebral vasodilatation, among others [32, 33].

Exposure to high-cholesterol diets inhibited hypoxia-induced liver glycogen depletion and decreased gluconeogenic gene expression. All of these changes induced by cholesterol indicated a dramatic decrease in the utilization of glucose for energy production. Cholesterol-rich diet consumption increased ketone body levels in liver tissues, a clear indication of free fatty acid oxidation and ketogenesis. The increased utilization of stored triglycerides manifested as significant dwindling of adipose tissue and increased liver size after 6 weeks of dietary treatment.

Ketone bodies protect tissues from reduced oxygen availability by multiple mechanisms, including reduced generation of ROS, improved mitochondrial efficiency, and activation of ATP-sensitive potassium channels (KATP) [35]. Ketone bodies act not only as metabolic substrates but also as metabolic modulators, protecting cells from hypoxic challenge [35]. Suzuki et al. [36] demonstrated that *β*-hydroxybutyrate prolonged survival time in rat models of hypoxia, anoxia, and global ischemia. Ketones decrease the O_2_ cost of ATP synthesis and offer an advantage over glucose as a fuel [35]. Therefore, it is likely that the shift to ketone body metabolism in cholesterol diet-fed mice was a protective adaptation to hypoxia-induced oxidative stress.

Unexpectedly, no increase in mRNA levels of the *β*-oxidation-related genes *PPAR-α* and *PGC-1α* was observed in cholesterol-fed mice; rather, the levels decreased. Oxidative breakdown of fatty acids consumes a large amount of oxygen in hypoxic conditions; therefore, it is possible that *PPAR-α* and *PGC-1α* expressions were inhibited due to synergistic activation of HIF-1*α* by hypoxia and cholesterol [37], resulting in the reprogramming of lipid metabolism to suppress excessive mitochondrial lipid catabolism through *β*-oxidation [37–39]. Decreased oxygen consumption in mitochondria serves as a safeguard for cell survival under hypoxia by inhibiting aberrant electron leakage from mitochondria and thereby preventing ROS production [38]. In addition to their suppression by HIF-1*α*, TNF-*α* and inflammation reduce *PGC-1α* and *PPAR-α* expression levels [40]. The increase in liver TNF-*α* ([Supplementary-material supplementary-material-1]) observed in cholesterol-fed mice may also explain the decreases in *PPAR-α* and *PGC-1α* mRNA levels.

Since *β*-oxidation genes were suppressed, we postulated that increased fatty acid flux may be the mechanism for the metabolic shift and increased ketone body levels. Therefore, it seems likely that this compensatory mechanism was responsible for the observed increase in ketone body levels in cholesterol-fed mice under hypoxic conditions. It is also worth noting that the increased fatty acid flux from adipose tissue to the liver in cholesterol-fed mice may have provided additional substrates for hepatocellular ketogenesis. In addition, an increase in AMPK activation and ACC inhibition was observed in cholesterol-fed mice. Consistently with liver AMPK activation, an increase in iNOS mRNA expression was observed in CH diet-fed mice. It has previously been shown that iNOS regulates AMPK activation [41]. Therefore, it is likely that the increase in liver iNOS expression led to activation of AMPK and thereby to increased ketogenesis. Since the iNOS protein is under the control of HIF-1*α*, its activation is connected to hypoxia and the need for a metabolic shift toward fat utilization.

Cholesterol treatment prevented hypoxia-induced hepatocellular death. In addition, cholesterol increased hepatocellular glucose secretion, indicating its role in the preservation of hepatocellular metabolic function.

In addition to the positive effect of increased ketone body levels, the induction of genes related to hypoxic adaptation by HIF-1*α* may prevent cell death. HIF-1*α* plays pivotal roles in cell survival and ATP generation during hypoxia. Moreover, the enhanced expression of *GLUT1* and *PDK1* mediated by HIF-1*α* in response to hypoxia represents a fundamental adaptation critical to cell viability and function [38, 42].

## 5. Conclusion

We now propose that cholesterol contributes to hypoxic adaptation in vivo and in vitro through induction of HIF-1*α* and iNOS expression. We postulate that AMPK activation by iNOS under increased fatty acid flux from adipose tissue to the liver enables hepatocellular ketogenesis by an AMPK-dependent compensatory pathway. This pathway involves increased free fatty acid uptake into mitochondria. This may allow ketone body production in spite of reduced *β*-oxidation gene expression. Increased ketogenesis protected CH diet-fed mice from hypoxia-induced hypoglycemia. In addition, cholesterol protected hepatocytes against hypoxia-mediated cell death probably by induction of HIF-1*α*-regulated genes related to hypoxic adaptation.

This suggested mechanism by which cholesterol contributes to metabolic shift, hypoxic adaptation, and survival may allow future development of therapeutic and nutritional strategies for prevention of hypoxic damage.

## Data Availability

The western blots and real-time PCR data used to support the findings of this study are available from the corresponding author upon request.

## Abbreviations

ACOX: Acyl-CoA oxidase
AMPK: AMP-activated protein kinase
KATP: ATP-sensitive potassium channels
BOX: Branched-chain acyl-CoA oxidase
CH: High-cholesterol diet
DMEM: Dulbecco's modified Eagle's medium
HIF-1*α*: Hematoxylin-eosin (H&E) hypoxia-inducible factor 1-*α*
HIG2: Hypoxia-inducible protein 2
LDs: Lipid droplets
PI: Propidium iodide
*β*-HB: *β*-Hydroxybutyrate.

## Supplementary Materials

Supplementary Materials Figure S1: effect of CH diet consumption for six weeks on mouse hepatic morphology. Representative histologic microphotographs of H&E-stained liver sections in mice fed with normal diet (A) or CH diet (B). Magnification ×20. Figure S2: effect of high-cholesterol diet (CH) and hypoxia (H) on mouse serum lipid profile. Effect of CH and H or their combination, as compared to the control, on triglyceride (A), total cholesterol (B), HDL cholesterol (C), and non-HDL cholesterol (D) levels in mouse sera. Values are expressed as *mean* ± *SE* (*n* = 8–10 for each group). Figure S3: effect of high-cholesterol diet (CH) consumption for three weeks and hypoxia (H) on mouse metabolic parameters. Effect of CH diet, hypoxia (H), or their combination (CH+H) as compared to the control on blood glucose levels (A), liver glycogen content (B), and serum ketone body levels (C). *n* = 8, *P* < 0.05. Figure S4: effect of high-cholesterol diet (CH) and hypoxia (H) on mouse liver damage and inflammation. Effect of CH diet and H or their combination, as compared to the control, on liver enzyme levels. SGOT (A) and SGPT (B) in mouse sera and on TNF-*α* (C) mRNA levels in mouse liver. Values are expressed as *mean* ± *SE* (*n* = 6–10 for each group). Table S1: diet composition. Table S2: RT PCR primers.
