# Supplementary material for: Cholesterol Prevents Hypoxia-Induced Hypoglycemia by Regulation of a Metabolic Ketogenic Shift
Source: Oxid Med Cell Longev. 2019 Sep 11;2019:5829357. doi: 10.1155/2019/5829357 (PMC6755303; doi:10.1155/2019/5829357)
Supplement: Supplementary Materials — Figure S1: effect of CH diet consumption for six weeks on mouse hepatic morphology. Representative histologic microphotographs of H&E-stained liver sections in mice fed with normal diet (A) or CH diet (B). Magnification ×20. Figure S2: effect of high-cholesterol diet (CH) and hypoxia (H) on mouse serum lipid profile. Effect of CH and H or their combination, as compared to the control, on triglyceride (A), total cholesterol (B), HDL cholesterol (C), and non-HDL cholesterol (D) levels in mouse sera. Values are expressed as mean ± SE (n = 8–10 for each group). Figure S3: effect of high-cholesterol diet (CH) consumption for three weeks and hypoxia (H) on mouse metabolic parameters. Effect of CH diet, hypoxia (H), or their combination (CH+H) as compared to the control on blood glucose levels (A), liver glycogen content (B), and serum ketone body levels (C). n = 8, P < 0.05. Figure S4: effect of high-cholesterol diet (CH) and hypoxia (H) on mouse liver damage and inflammation. Effect of CH diet and H or their combination, as compared to the control, on liver enzyme levels. SGOT (A) and SGPT (B) in mouse sera and on TNF-α (C) mRNA levels in mouse liver. Values are expressed as mean ± SE (n = 6–10 for each group). Table S1: diet composition. Table S2: RT PCR primers. [file 5829357.f1.pptx]

## Slide 1
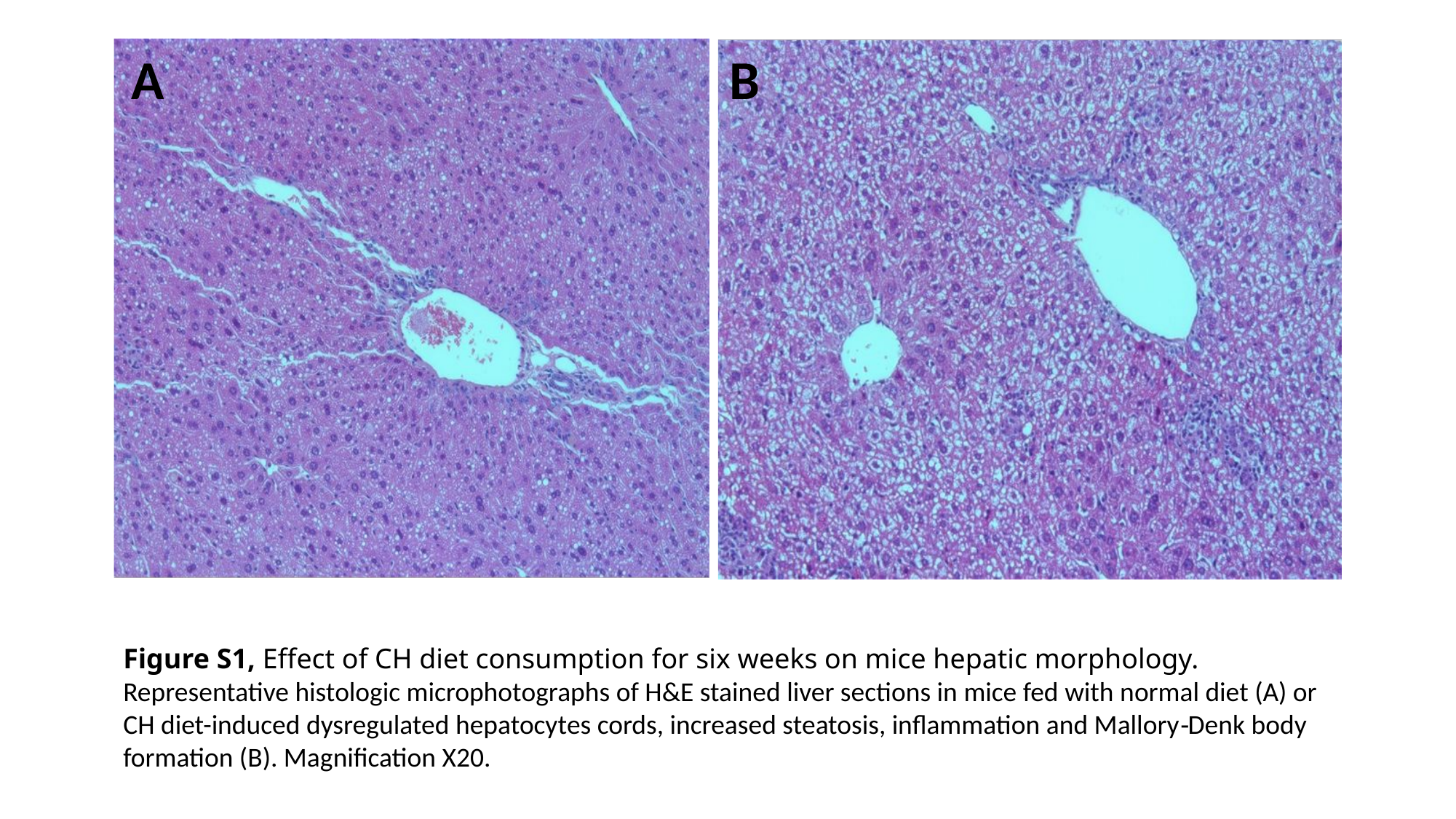

A
B
Figure S1, Effect of CH diet consumption for six weeks on mice hepatic morphology.
Representative histologic microphotographs of H&E stained liver sections in mice fed with normal diet (A) or CH diet-induced dysregulated hepatocytes cords, increased steatosis, inflammation and Mallory‐Denk body formation (B). Magnification X20.

## Slide 2
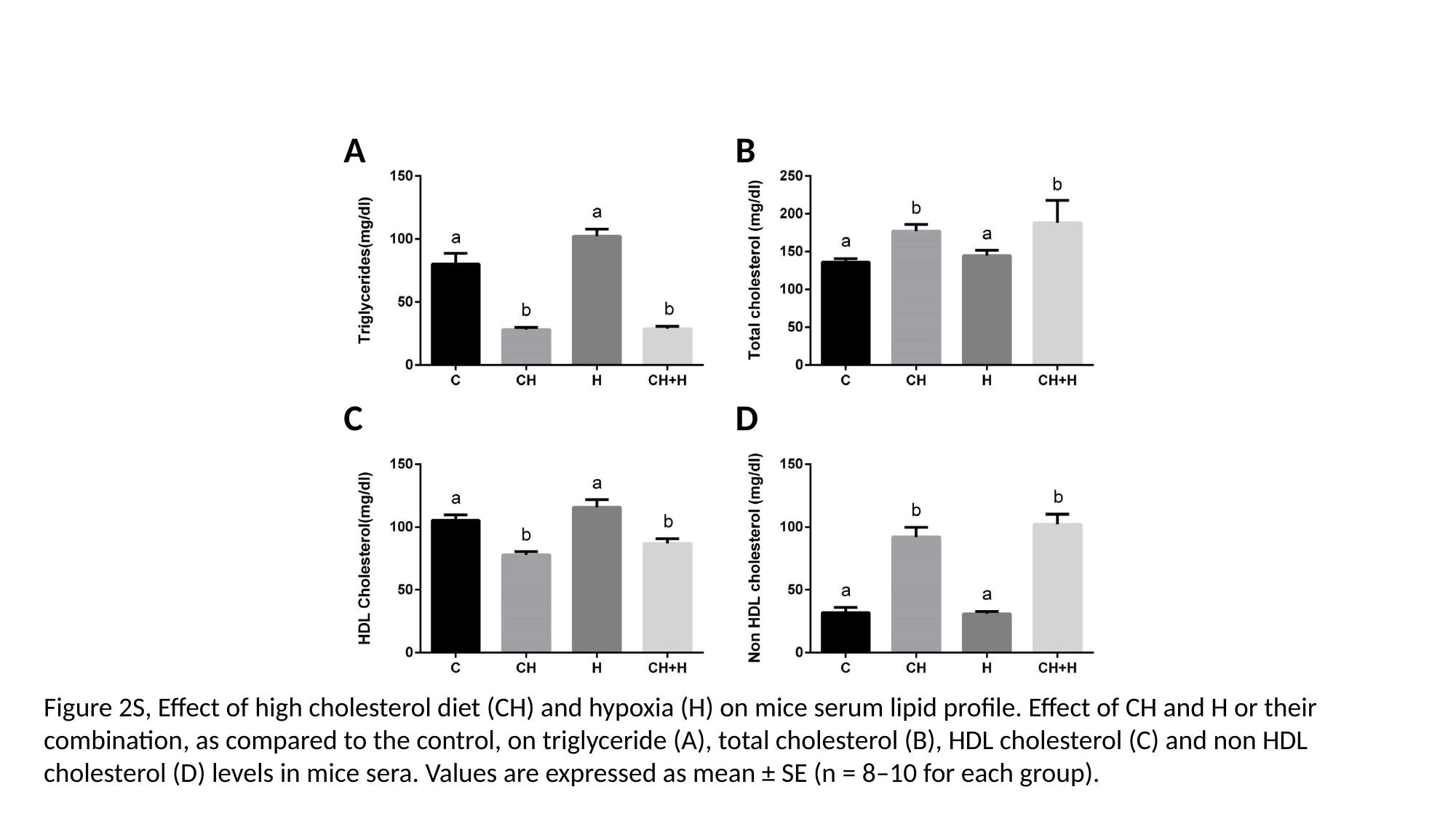

#
A
B
C
D
Figure 2S, Effect of high cholesterol diet (CH) and hypoxia (H) on mice serum lipid profile. Effect of CH and H or their combination, as compared to the control, on triglyceride (A), total cholesterol (B), HDL cholesterol (C) and non HDL cholesterol (D) levels in mice sera. Values are expressed as mean ± SE (n = 8–10 for each group).

## Slide 3
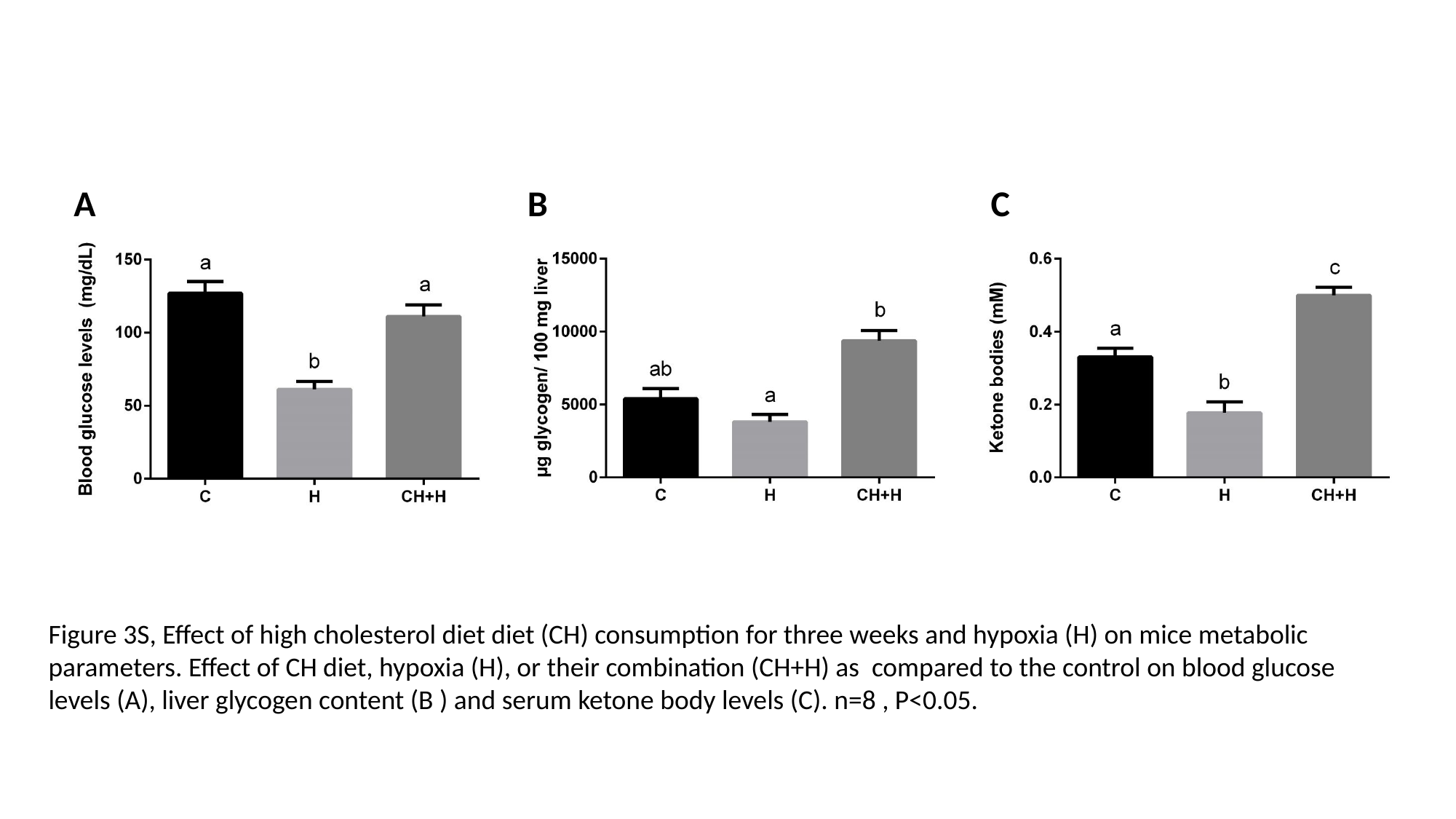

#
A
B
C
Figure 3S, Effect of high cholesterol diet diet (CH) consumption for three weeks and hypoxia (H) on mice metabolic parameters. Effect of CH diet, hypoxia (H), or their combination (CH+H) as compared to the control on blood glucose levels (A), liver glycogen content (B ) and serum ketone body levels (C). n=8 , P<0.05.

## Slide 4
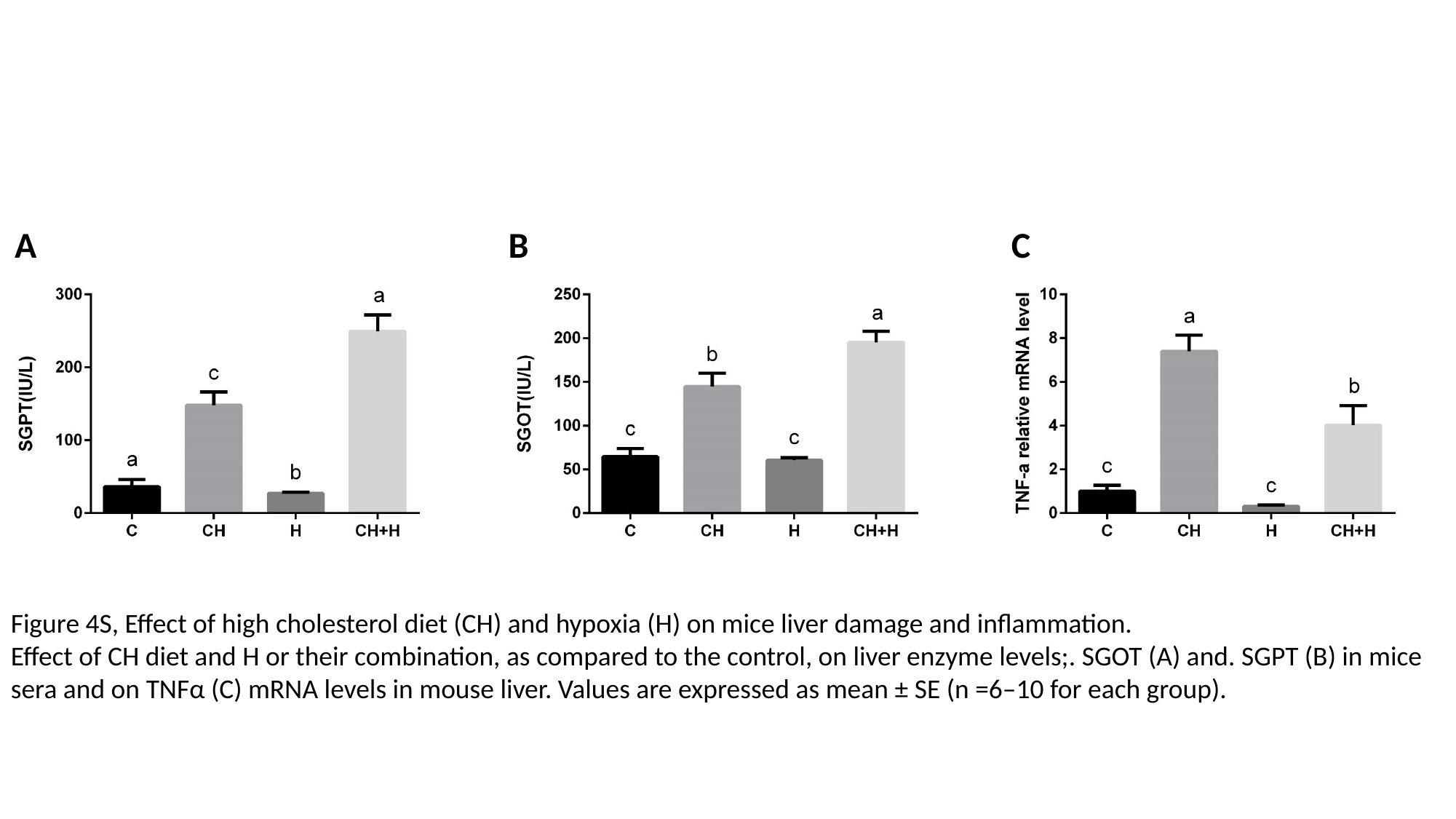

#
A
B
C
Figure 4S, Effect of high cholesterol diet (CH) and hypoxia (H) on mice liver damage and inflammation.
Effect of CH diet and H or their combination, as compared to the control, on liver enzyme levels;. SGOT (A) and. SGPT (B) in mice sera and on TNFα (C) mRNA levels in mouse liver. Values are expressed as mean ± SE (n =6–10 for each group).

## Slide 5
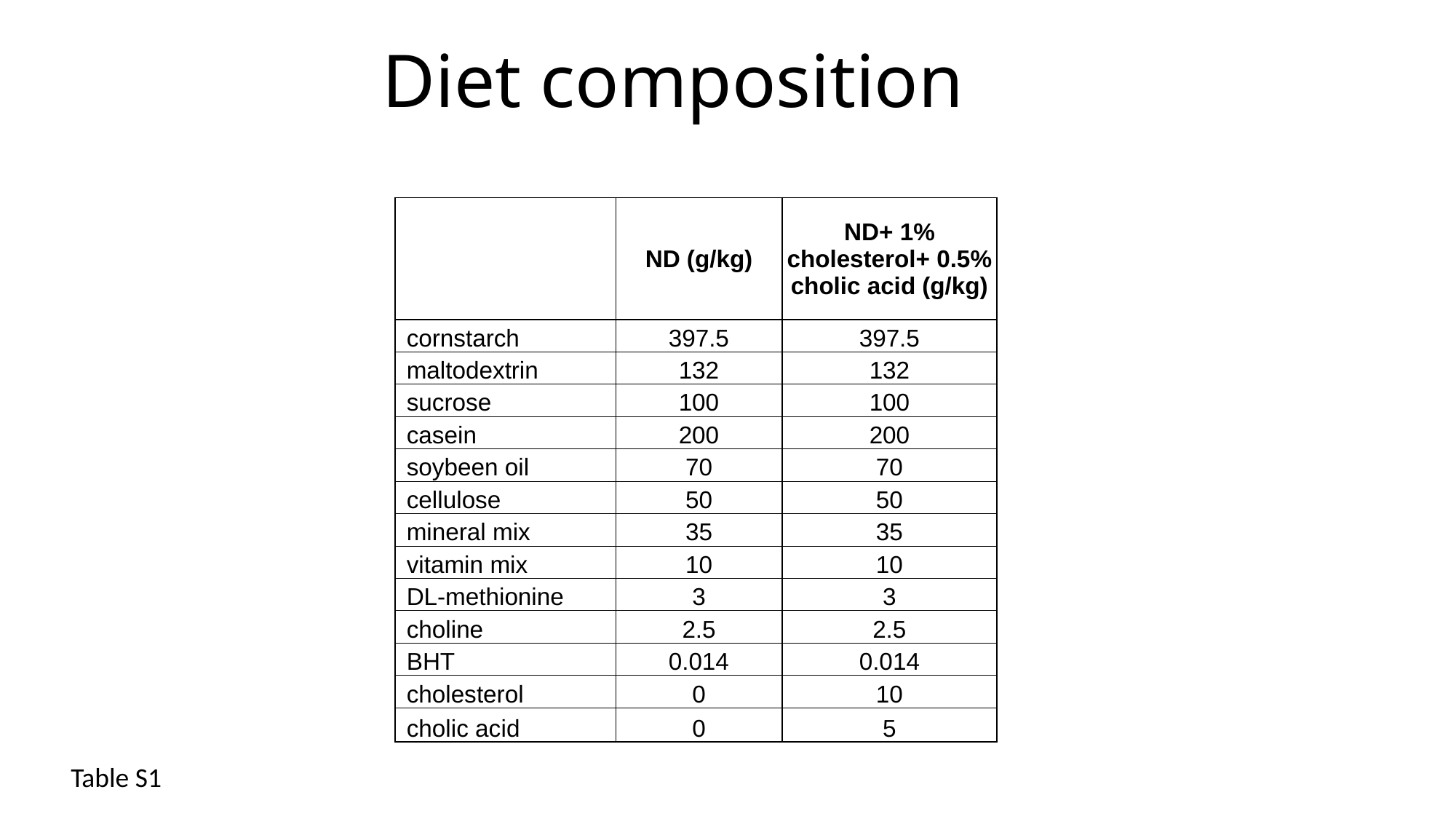

Diet composition
| | ND (g/kg) | ND+ 1% cholesterol+ 0.5% cholic acid (g/kg) |
| --- | --- | --- |
| cornstarch | 397.5 | 397.5 |
| maltodextrin | 132 | 132 |
| sucrose | 100 | 100 |
| casein | 200 | 200 |
| soybeen oil | 70 | 70 |
| cellulose | 50 | 50 |
| mineral mix | 35 | 35 |
| vitamin mix | 10 | 10 |
| DL-methionine | 3 | 3 |
| choline | 2.5 | 2.5 |
| BHT | 0.014 | 0.014 |
| cholesterol | 0 | 10 |
| cholic acid | 0 | 5 |
Table S1

## Slide 6
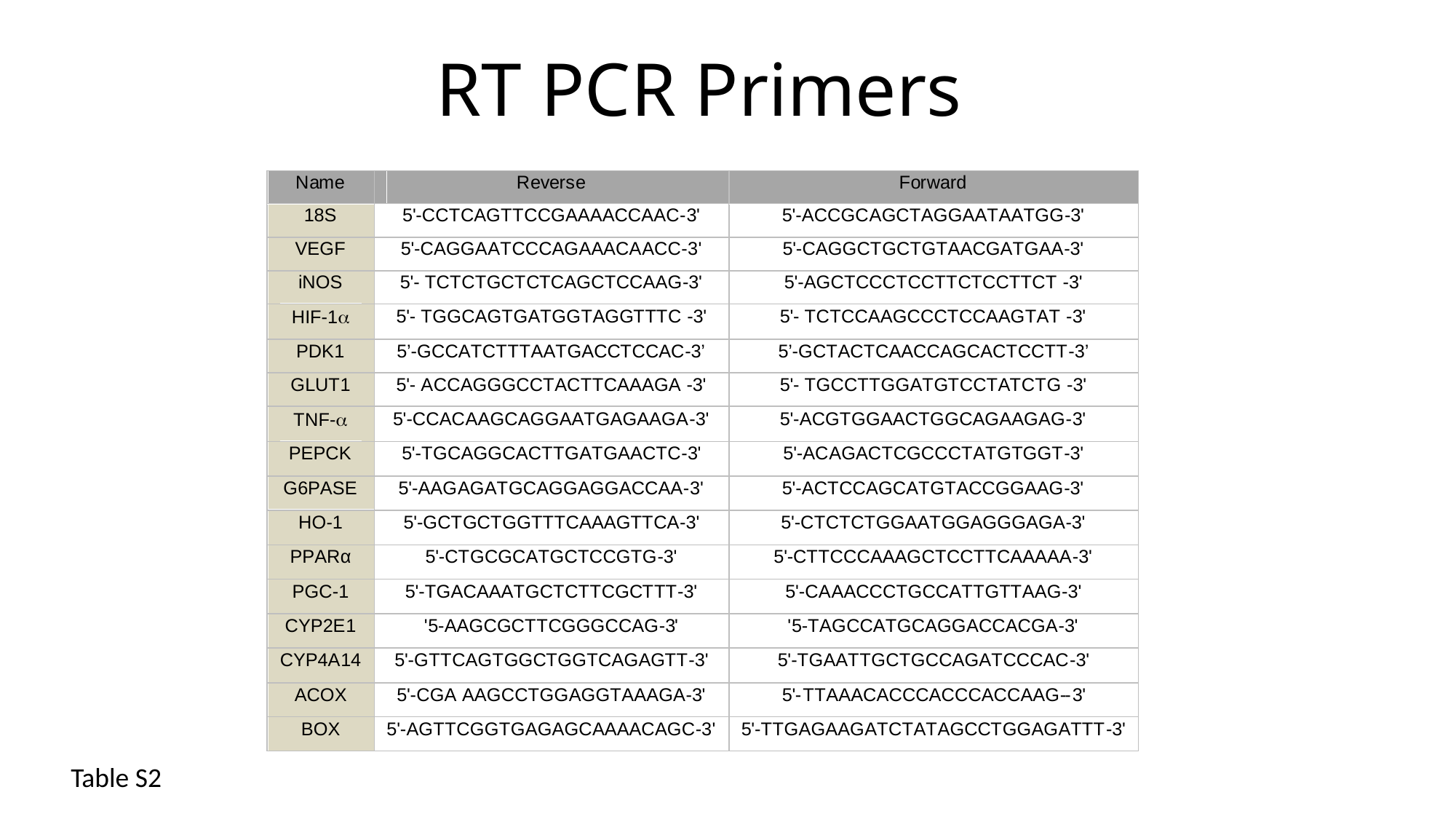

RT PCR Primers
Table S2
